# The effect of cigar, cigarillo and pipe smoking on lung cancer risk and screening eligibility

**DOI:** 10.1038/s41416-026-03578-9

**Published:** 2026-08-07

**Authors:** Simran Vaja, Jennifer L. Dickson, Carolyn Horst, Monica L. Mullin, Amyn Bhamani, Andrew Creamer, Helen Hall, Chuen R. Khaw, Ruth Prendecki, Priyam Verghese, Sophie Tisi, John McCabe, Esther Arthur-Darwka, Neal Navani, Anand Devaraj, Arjun Nair, SUMMIT consortium, Sam M. Janes, Allan Hackshaw

**Affiliations:** 1 Cancer Research UK and UCL Cancer Trials Centre, University College London, London, UK; 2 West Suffolk Hospital Foundation Trust, Bury St Edmunds, UK; 3 Lungs For Living, UCL Respiratory, Department of Medicine, University College London, London, UK; 4 King’s College London and Guy’s & St Thomas’ NHS Foundation Trust, London, UK; 5 Division of Respiratory Medicine, Cumming School of Medicine, University of Calgary, Calgary, AB, Canada; 6 Imperial College Healthcare NHS Trust, London, UK; 7 Epsom and St Helier Hospitals NHS Trust, Epsom, UK; 8 University College London Hospitals NHS Foundation Trust, London, UK; 9 Mid and South Essex NHS Foundation Trust, Basildon, UK; 10 National Heart and Lung Institute, Imperial College, London, UK; 11 Royal Brompton and Harefield Hospitals, London, UK

**Keywords:** Cancer, Epidemiology

## Abstract

**Background:**

Smokers are eligible for lung cancer screening using low dose CT (LDCT) if they have a sufficiently high risk of lung cancer. In England, for example, people are eligible if their estimated risk using either of two validated models (PLCO_m2012_ and Liverpool Lung Project) exceeds a certain threshold. Risk is often estimated using only cigarette consumption (duration and number per day), yet many smokers also use other cancer-causing tobacco products.

**Methods:**

SUMMIT is a prospective cohort study of a LDCT screening service. We evaluated the impact of cigars, pipes, and cigarillos on lung cancer risk.

**Results:**

Among 13,000 participants who were current/former cigarette smokers, 511 (1 in 25) were also regular users of cigars, pipes, or cigarillos. The 5-year cumulative lung cancer incidence in people who currently smoked both types of tobacco products was 8.85%, versus 5.74% for cigarette only current smokers. Among these 511 participants, the percentage whose risk was below the screening eligibility threshold ignoring other tobacco products which then exceeded the threshold including these products was 0.6% using LLPv2, and 8.2% using PLCO_m2012_.

**Conclusions:**

All tobacco consumption should be considered when determining screening eligibility to avoid missing those who can benefit from LDCT.

**Clinical trial registration:**

Clinicaltrials.gov no: NCT03934866.

## Background

Globally, lung cancer was the most common cancer type in 2022 (12.4% of all cancers), and the leading cause of cancer death (1.8 million deaths annually) [1]. Population screening for lung cancer using low dose CT (LDCT) is recommended in the US [2] and programmes are in place in other countries such as the UK that has rapidly increasing coverage with full roll out and completion of the prevalence screen planned for 2030 [3]. Unlike cancer screening for breast, cervical and bowel, that are based on age alone, LDCT screening is only cost-effective in higher risk people (i.e. smokers) because of the low incidence in never-smokers or former smokers who quit many years ago. The US Preventive Services Task Force (USPSTF 2013) recommends screening for adults aged 50 to 80 years who have a 20 pack-year smoking history and currently smoke or have quit within the past 15 years [2]. However, ageing alone increases absolute lung cancer risk beyond 15 quit-years, so there is an argument that such people should not be exempt from screening [4].

Risk-based targeted cancer screening improves efficiency by increasing the number of people with the cancer of interest who are eligible while reducing the false-positive rate [5, 6]. Risk prediction models use demographic or behavioural characteristics or biomarkers. The most well-known model for lung cancer is the Prostate, Lung, Colorectal and Ovarian (PLCO)_m2012_ [7]. Other models have been developed for specific countries, for example in the UK: the Liverpool Lung Project (LLP), the UK Biobank Prospective Cohort Study (UK Biobank), and CanPredict (QResearch and Clinical Practice Research Datalink national primary care datasets) [8–11]. In the UK, the Lung Cancer Screening Programme uses both PLCO_m2012_ and the LLP version 2.

Quantifying tobacco exposure is a crucial element of lung cancer risk: whether by the number of cigarettes smoked or pack-years (a combination of cigarette consumption and duration). Tobacco exposure can come from cigarettes, cigars, cigarillos, pipes and smokeless tobacco. However, researchers have often specifically only considered cigarette use when deriving and applying lung cancer risk models. This was because the prevalence of tobacco products other than cigarettes in the studies used to develop risk models was low, particularly in the US (PLCO_m2012_).

Systematic reviews clearly show that people who smoke cigars and pipes, with or without concurrent cigarette use, have a significantly higher risk of lung cancer compared to never-smokers [12, 13]. Screening eligibility is sensitive to tobacco exposure, so it is important that risk is accurately estimated in populations that use multiple tobacco products. Otherwise, people who are heavy users of cigars or pipes, alongside cigarette use, could be misclassified as having insufficient risk and therefore be ineligible for LDCT. Cigars and pipes are used in the UK and several European countries. We used the SUMMIT study to examine the effect on lung cancer risk and LDCT eligibility.

## Methods

SUMMIT is a large prospective cohort study designed to examine the performance of delivering an LDCT screening service to a high-risk population in London and to validate a multi-cancer early detection blood test (ClinicalTrials.gov NCT03934866) [14, 15]. Individuals aged 55 to77 registered with participating GP surgeries and coded as being a smoker within the past 20 years were invited to attend a Lung Health Check at one of four LDCT scanning sites across North and East London. At this assessment, individuals were eligible for the study and LDCT if they met either the USPSTF 2013 criteria (i.e. at least 30 pack-year history and if a former smoker they had stopped within 15 years), or they had a PLCO_m2012_ risk estimate of ≥1.3%. For both criteria only cigarette consumption was considered [16]. Eligible individuals were offered a same day LDCT, and they had two subsequent annual visits for a lung health check and one or two more annual LDCT scans, unless they required additional scans for nodule management.

Individuals were asked at baseline about their cigar, cigarillo and pipe smoking habits. They were asked how often they smoked these products: occasionally (less than weekly) or regularly (at least once per week). For those who regularly smoked cigars, cigarillos, or pipes, they were asked whether they were current or former users, at what age they started smoking them, and the amount per week. This information was in addition to cigarette habits.

The PLCO_m2012_ model was developed using smokers in the control arm of the PLCO trial in the US, which produces a predicted risk of lung cancer over the next 6 years. The LLPv2 risk model was developed in England and it produces a 5-year risk. It should be noted that although the original LLP risk model only included cigarettes it was later revised to include other smoking products in LLPv2 [9]. This model incorporating multiple tobacco products was used to determine eligibility for the randomised UKLS screening trial of LDCT and meant for eligibility assessment in the UK screening programme. Hence some other groups have indeed allowed for multiple tobacco products when using certain risk models. The aim of our paper here is to recommend that this is always done, especially in places where the use of products other than cigarettes is relatively high.

Lung cancer was diagnosed as per standard clinical practice. Individuals with an abnormal LDCT had cancer investigations at a local secondary care hospital following a referral from the study. Cancer diagnoses were also collected from the National Cancer Registration and Analysis Service (NCRAS), an established national registry in England to capture cancers diagnosed outside of the study. The date of the last NCRAS download was 9 June 2025 and if SUMMIT participants were not listed in this dataset, they were considered to be alive and cancer-free up to that timepoint. Time to lung cancer diagnosis was calculated from the date of entry to SUMMIT. Individuals without lung cancer were censored at the date of the NCRAS download.

We conducted two main analyses. The first examined the association between lung cancer and different combinations of tobacco products (distinguishing former and current cigarette users). A Cox proportional regression produced hazard ratios adjusted for sex, ethnicity, age, BMI, IMD rank, education, history of COPD, bronchitis or emphysema, baseline airflow obstruction, previous cancer, and family history of lung cancer. A backward selection method (*p* = 0.10 level) was applied to produce a subset of factors for a final set of adjusted hazard ratios (HR). Cumulative incidence curves, adjusted for these factors, were presented for various combinations of tobacco products.

In the other analysis, we re-calculated both PLCO_m2012_ and LLPv2 to determine the number of individuals whose lung cancer risk increased when including cigars/cigarillos/pipes. Cigars and pipes were converted to number of cigarette equivalents per day, using a published conversion in the UK health service [17]. In the UK lung screening programme individuals are eligible if either their PLCO_m2012_ 6-year risk is ≥1.51%, or their LLPv2 5-year risk is ≥2.5%. We examined the number of participants who smoked cigars, cigarillos or pipes whose estimated risk was below these thresholds excluding these products in the risk estimation, and the number whose risk exceeded this threshold when these products were included. This analysis was possible because participants could be eligible for the SUMMIT study using the USPSTF criteria alone (so their PLCO_m2012_ risk could be <1.3%).

Stata (version 19.5) and R (version 4.4.1) were used for statistical analysis.

## Results

**Fig. 1:**
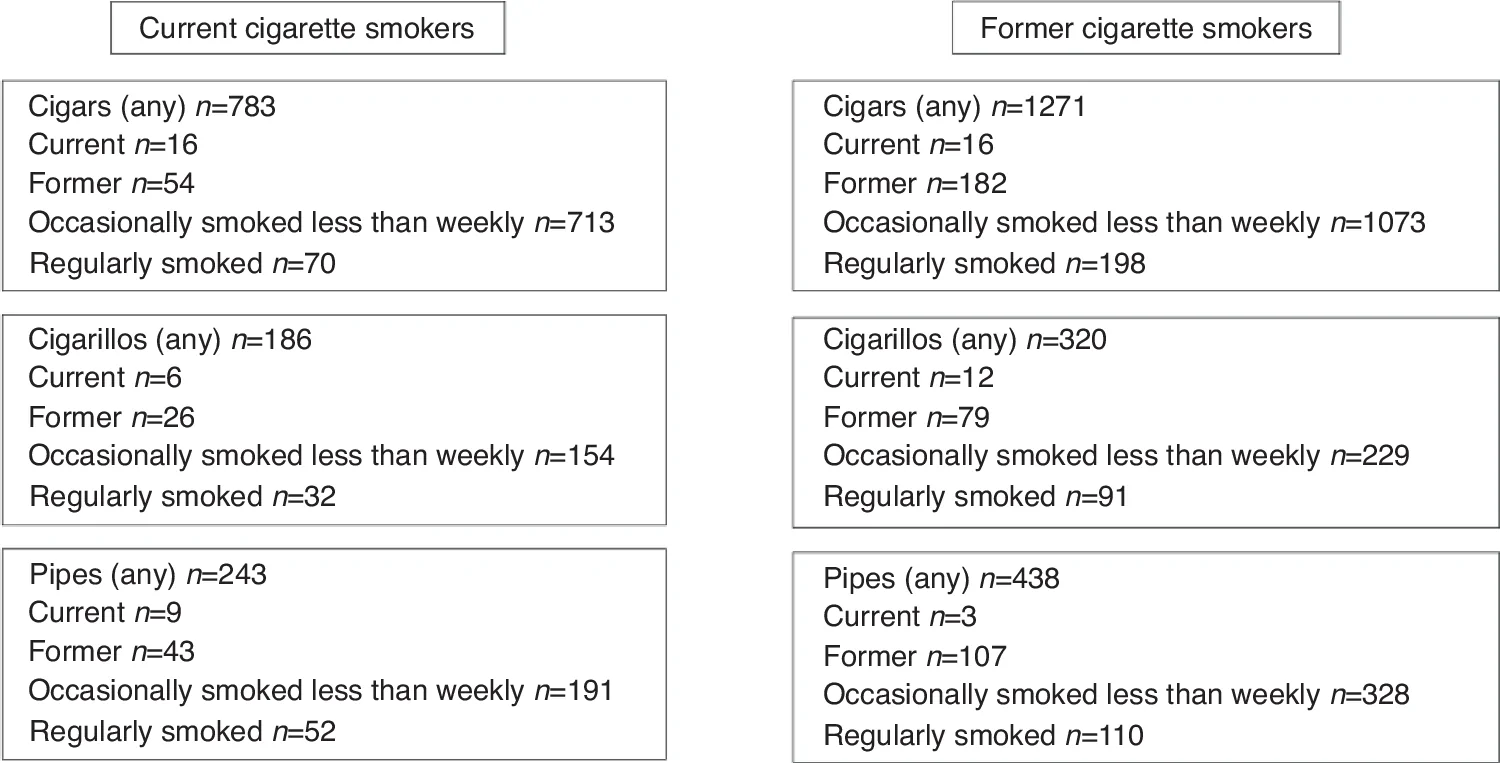
The number of people who smoked cigars, cigarillos or pipes in the SUMMIT participants, classified according to whether they were also current or former cigarette smokers.

18,581 people attended the Lung Health Check, of which 13,035 were eligible (according to PLCO_m2012_ or LLPv2 criteria) and consented to participate in the study. We excluded 28 who had an active cancer at enrolment as this might affect the risk of lung cancer and another 7 ineligible for other reasons; leaving 13,000 for the analysis. They were recruited between April 8, 2019, and May 14, 2021, of which 12,773 were analysed for evaluating LDCT [18]. After a median of 64 months follow up, 654 participants were diagnosed with lung cancer. Figure 1 shows cigar, cigarillo and pipe use within the SUMMIT study. Former cigarette smokers were more likely to have used cigars, cigarillo, and pipes (1,271/6,640 (19.1%), 320/6,640 (4.8%), and 438/6,640 (6.6%), respectively) than current cigarette smokers (783/6,360 (12.3%), 186/6,360 (2.9%), and 243/6,360 (3.8%), respectively). Most participants who currently or formerly smoked cigarettes had occasionally smoked these other types of tobacco less than weekly. Regular smokers of pipes were the most common, followed by cigarillos, and cigars (21.4%, 17.2%, and 8.9%, respectively) among current cigarette smokers, whereas for former cigarette smokers, regular cigarillo use was most common, followed by pipes, and cigars (28.4%, 25.1%, and 15.6%, respectively). Among 511 regular cigar, cigarillo, or pipe smokers (either current or past users) there were 24 lung cancers; compared to 332 lung cancers among the 6217 current cigarette only smokers, and 298 cancers among the 6272 former cigarette only smokers.

Table 1 summarises the characteristics of the SUMMIT participants. 2,299 (17.7%) of participants were ever smokers of cigars or cigarillos, 681 (5.2%) were ever smokers of pipes, and 12,489 (96.1%) were never-smokers of any of these types and only smoked cigarettes. The majority of smokers of cigars, cigarillos, or pipes were male, compared with cigarette only smokers where the sex distribution was more equal. Cigar, cigarillo or pipe smokers were more common amongst those who completed further education and those in the lowest two deprivation quintiles (the highest socio-economic status). Ever smokers of pipes were slightly older than ever smokers of cigars or cigarillos, or cigarette only smokers.

**Table 1:** Characteristics of smokers (there is overlap between the 2299 cigar/cigarillo and 681 pipe smokers).

		Ever smokers of cigars or cigarillos (*N* = 2299)	Ever smokers of pipes (*N* = 681)	Non-smokers of cigars, cigarillos, or pipes, i.e. cigarettes only (*N* = 12,489)
**Lung cancer**	No	2184 (95.0%)	646 (94.9%)	11859 (95.0%)
	Yes	115 (5.0%)	35 (5.1%)	630 (5.0%)
**Sex**	Female	290 (12.6%)	38 (5.6%)	5493 (44.0%)
	Male	2009 (87.4%)	643 (94.4%)	6996 (56.0%)
**Ethnicity**	White	2059 (89.6%)	604 (88.7%)	10903 (87.3%)
	Black	98 (4.3%)	22 (3.2%)	666 (5.3%)
	Asian/other	142 (6.2%)	55 (8.1%)	920 (7.4%)
**Education**	Finished school at or before 15 years	869 (37.8%)	215 (31.6%)	4987 (39.9%)
	Completed CSCs, O-levels, or equivalent	494 (21.5%)	126 (18.5%)	2995 (24.0%)
	Completed A-levels or equivalent	224 (9.7%)	69 (10.1%)	1356 (10.9%)
	Completed further education but not a degree	229 (10.0%)	75 (11.0%)	1041 (8.3%)
	Completed a bachelor’s degree or equivalent	344 (15.0%)	134 (19.7%)	1501 (12.0%)
	Completed a further degree, e.g. masters/PhD	139 (6.1%)	62 (9.1%)	609 (4.9%)
**Index of Multiple Deprivation quintile (measure of social deprivation)**	1 (most deprived)	671 (29.2%)	190 (27.9%)	4037 (32.3%)
	2	675 (29.4%)	195 (28.6%)	3740 (30.0%)
	3	384 (16.7%)	127 (18.7%)	2174 (17.4%)
	4	398 (17.3%)	121 (17.8%)	1795 (14.4%)
	5 (least deprived)	150 (6.5%)	41 (6.0%)	607 (4.9%)
	Missing	21 (0.9%)	7 (1.0%)	136 (1.1%)
**Age**	Mean (SD) Median (IQR)	66.2 (6.0) 66 (61-71)	67.7 (5.7) 68 (63-72)	65.3 (6.1) 65 (60-70)
**BMI, kg/m**^**2**^	Mean (SD) Median (IQR)	28.5 (5.1) 28 (25-31.3)	28.5 (5.1) 28 (25.1-31.4)	28.0 (5.4) 27.4 (24.2-30.9)
**Cigarettes smoked a day**	Mean (SD) Median (IQR)	22.8 (11.1) 20 (15-25)	23.1 (11.9) 20 (15-25)	21.1 (10.2) 20 (15-20)

The smoking habits of the *n* = 511 regular users of cigars, cigarillos, or pipes are summarised in Supplementary Tables 2, 3. The median number of cigarettes smoked per day was similar between those who smoked cigarettes only and those who also smoked other products (about 20 per day). However, current users of cigars, cigarillos, or pipes had smoked cigarettes for slightly longer than current cigarette only smokers (median 50 vs 47 years, Supplementary Table 3).

**Fig. 2:**
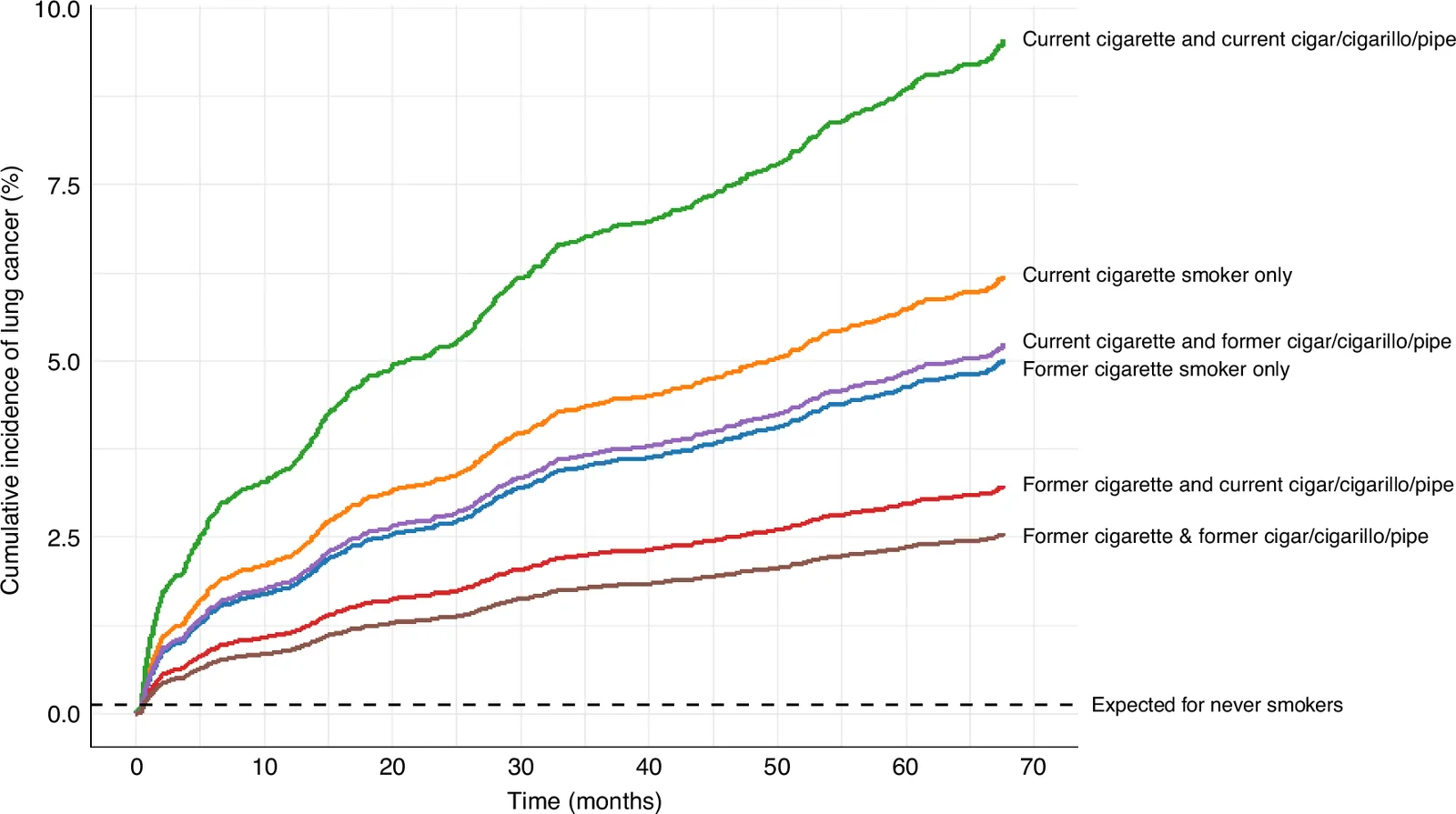
Adjusted cumulative incidence curves for current regular or former regular smokers of cigars, pipes, or cigarillos (adjusted for age, BMI, ethnicity, family history of lung cancer, previous history of cancer, education level and baseline airflow obstruction); time since study entry. The apparent “waves” in the curves are partly due to the timing of the screening rounds. The horizontal dashed line shows the expected 5-year cumulative lung cancer incidence for never-smokers as an approximate reference (not observed in the study), acknowledging that in reality incidence would also be a curve that increases (by age and time) and not be a straight line.

The backwards selection regression produced age, BMI, ethnicity, family history of lung cancer, previous history of cancer, education level and baseline airflow obstruction as the set of factors associated with lung cancer and used in the regression modelling. Figure 2 shows the adjusted incidence of lung cancer according to current and former use of cigarettes or cigars/cigarillos/pipes. The highest incidence of lung cancer is, as expected biologically, seen in people who currently smoke both cigarettes and cigars/cigarillos/pipes, with adjusted hazard ratio 1.58 (95%CI 0.39-6.34) compared to current cigarette only smokers (Table 2). The 2-year and 5-year incidence in people who smoke both types of tobacco products were 5.21% (95% CI 0–12.0) and 8.85% (95% CI 0–20.10), respectively, higher than the corresponding risks for cigarette only current smokers, 3.34% (95% CI: 2.9–3.79) and 5.74% (95% CI 5.07–6.40). For comparison, the expected 2- and 5-year risk in never-smokers (not included in the SUMMIT study) would be about 0.13% and 0.23% (applying a relative risk of lung cancer of 25, current vs never smokers); comparable to the observed cumulative risk of lung cancer by age 75 in UK male never-smokers, 0.2% [19].

**Table 2:** Association between tobacco product use and lung cancer risk.

Variable	Unadjusted HR (95% CI)	Adjusted HR (95% CI)^a^
Cigarette smoker only: Current (*n* = 6217)	Reference	Reference
Cigarette smoker only: Former (*n* = 6272)	0.89 (0.76-1.04)	0.80 (0.67-0.95)
Cigar/pipe/cigarillo smoker: Current^b^ + **current** cigarette smoker (*n* = 31)	1.81 (0.58-5.63)	1.58 (0.39-6.34)
Cigar/pipe/cigarillo smoker: Current^b^ + **former** cigarette smoker (*n* = 31)	0.57 (0.08-4.06)	0.51 (0.07-3.63)
Cigar/pipe/cigarillo smoker: Former^b^ + **current** cigarette smoker (*n* = 112)	0.99 (0.44-2.22)	0.84 (0.35-2.03)
Cigar/pipe/cigarillo smoker: Former^b^ + **former** cigarette smoker (*n* = 337)	0.78 (0.46-1.33)	0.40 (0.20-0.81)

^a^Adjusted for family history, ethnicity, age, BMI, previous cancer, education and airflow obstruction (the only factors that came from a stepwise selection model based on all factors, see Methods).

^b^Regular current or regular former smokers of cigars, pipes, or cigarillos.

Current cigarette smokers who were former users of cigars/cigarillos/pipes had a similar incidence as former cigarette only smokers (Fig. 2), while the HR when compared to current cigarette only smokers was 0.84 (95%CI 0.35–2.03); Table 2. The HRs among former cigarette smokers indicated a decreased risk for either current or former users of cigars/cigarillos/pipes, 0.51 (95%CI 0.07–3.63) and 0.40 (95%CI 0.20–0.81), respectively. These probably reflect that people who smoke cigars/cigarillos/pipes consume fewer of these than cigarettes, and this contributes more to their risk than past cigarette consumption. Whereas among current cigarette smokers who either currently or formerly smoked cigars/cigarillos/pipes their risk is more influenced by their cigarette consumption. However, it is important to remember that the HRs have current cigarette only smokers as the reference group, so a HR < 1 still means that the risk of lung cancer will be higher than in never-smokers (not represented in our study). In Fig. 2, all groups, whether current or former users of cigars/cigarillos/pipes, have incidence substantially higher than expected for never-smokers.

Among all 13,000 screened individuals, 12,958 by PLCO_m2012_ and 12,997 by LLPv2 would be eligible using the respective thresholds (≥1.5% and ≥2.5%) for the national lung cancer screening programme, ignoring non-cigarette tobacco products.

Focussing on the 511 who were regular users of cigars/cigarillos/pipes, n = 3 of these 511 participants (0.6%) would have a LLPv2 risk value that would change from below to above the threshold for screening eligibility if these products are included (Table 3). The mean 5-year risk was 12.0% (95%CI 11.3-12.7) without cigars/cigarillos/pipes and 13.0% (95%CI 12.2-13.7) including these products, i.e. 8% higher (13/12%).

**Table 3:** The effect on screening eligibility by including cigars, cigarillos and pipes in risk estimated using PLCO_2012_ or LLPv2.

	No. of people	Percentage (number) of people whose risk changes from below to above the threshold for screening eligibility	McNemar’s test for a paired comparison of with vs without cigars, cigarillos or pipes^a^
**PLCO**_**2012**_ **– threshold 6-year risk** ≥ **1.51%**			
Cigar, cigarillo or pipe smokers	511	8.2% (42/511) 95%CI 6.0-10.9%	*P* < 0.001
Cigar, cigarillo or pipe smokers, who are current cigarette smokers	143	0.7% (1/143) 95% CI 0.02-3.8%	*P* = 0.32
Cigar, cigarillo or pipe smokers, who are former cigarette smokers	368	11.1% (41/368) 95%CI 8.1-14.8%	*P* < 0.001
All SUMMIT participants	13,000	0.3% (42/13,000) 95% CI 0.2-0.4%	*P* < 0.001
**LLP version 2 - threshold 5-year risk** ≥ **2.5%**			
Cigar, cigarillo or pipe smokers	511	0.6% (3/511) 95%CI 0.1-1.7%	*P* = 0.08
Cigar, cigarillo or pipe smokers, who are current cigarette smokers	143	All were already above threshold	-
Cigar, cigarillo or pipe smokers, who are former cigarette smokers	368	0.8% (3/368) 95%CI 0.2-2.4%	*P* = 0.08
All SUMMIT participants	13,000	0.02% (3/13,000) 95%CI 0.005-0.07%	*P* = 0.08

The LLPv2 risk model applied above is calculated with and without cigars, cigarillos and pipes just to see what the effect on risk is. However, the publication that reported LLP version 2 does indicate that these other products can be included [9].

^a^Based on a 2×2 table of below or above the risk threshold and risk with or without these other tobacco products.

Using PLCO_m2012_, 42 of the 511 participants (8.2%) would have a risk value that would change from below to above the threshold for screening eligibility; or 11.1% (41/368) among those who were former cigarette smokers (Table 3). The mean 6-year risk was 4.5% (95%CI 4.1–4.9) without cigars/ cigarillos/pipes and 5.2% (95%CI 4.8–5.6) including these products. i.e. 16% higher (5.2/4.5%). The larger effect was seen with PLCO_m2012_ because this uses both duration of smoking and number of cigarettes (or cigarette equivalents that we use here), but LLPv2 only uses duration.

The above analyses were based on 13,000 individuals who were eligible and consented to the SUMMIT study. However, among the total 18,581 who attended the Lung Health Check, there were 5451 individuals whose risk of lung cancer was below the thresholds for PLCO_m2012_ or LLPv2, making them ineligible to enter the study; and they had information that could be used to calculate risk using these two models. By including their use of cigars and pipes, the percentage whose risk would have exceeded the thresholds become 0.4% (21/5451; or 1 in 260) for PLCO_m2012_, and 1.2% (63/5451; or 1 in 86) for LLPv2. Because these 5451 were not part of the SUMMIT study, we did not have their lung cancer status.

## Discussion

In our large lung cancer screening study, we show that there could be smokers who are ineligible for LDCT screening when using PLCO_m2012_ or LLPv2 based on cigarette consumption alone, who would become eligible if their cigar, cigarillo or pipe consumption is also accounted for. Many of these are due to being marginally below the thresholds. These individuals would not be offered screening and therefore be disadvantaged simply due to a statistical calculation and a decision to only focus on cigarette use.

About 1 million individuals are expected to be invited annually to the English lung cancer screening programme. The screening questions for eligibility and risk assessment include consumption of all tobacco products and the risk algorithm can indeed include these products if they are selected, although it is unclear how widely this is done across the country. To estimate the impact, we assume a conservative approach that these products are not allowed for at all. Assuming the prevalence of regular users of cigars, cigarillos or pipes in those invited for eligibility assessment is similar to SUMMIT (3.9%, 511/13,000), this represents 39,000 individuals of which potentially up to 3200 might only become eligible if those other products are used to estimate risk (8.2% in SUMMIT whose PLCO_m2012_ risk increased from below to above the threshold). We also had another estimate of this from individuals whose risk did not meet either USPSTF or PLCO_m2012_ criteria so were not eligible to enrol into the SUMMIT study. If their cigar and pipe habits were included in the risk estimation 1 in 260 or 1 in 86 would then exceed the risk thresholds for PLCO_m2012_ and PLCO_m2012_ respectively. Although these percentages appear small, they represent many people on a population scale. Further estimates of this could be obtained from a cohort of people who would be invited for screening.

There are also likely to be people with heavy exposure (including long duration) of other tobacco products alone (i.e. they do not or rarely use cigarettes as well) and are at high risk. It would be very interesting to see how many such individuals would be ineligible for screening by ignoring these products. Unfortunately, we cannot determine this in the SUMMIT study because eligibility only included cigarettes so those other users were automatically ineligible. The UK screening programme should be able to report these data.

In populations where the prevalence of tobacco products other than cigarettes is very low, incorporating consumption of those products in risk models is unlikely to affect the eligibility for most smokers, so current LDCT eligibility in the US, for example, is appropriate. Cigar and pipe use was very uncommon in the PLCO study used to develop the PLCO_m2012_ score, so there were likely very few lung cancers resulting from these. With limited data on cigar and pipe use it is understandable that the PLCO_m2012_ score focused on cigarettes only.

However, in several other countries (e.g. UK, Spain, Germany, Italy, Mexico and Brazil) cigars or pipes are used by many people. As lung cancer screening programmes are established or continue, the definition of high risk should allow for all tobacco products.

There is established evidence on the harms of cigars and pipes. The International Agency for Research on Cancer (IARC) monograph on tobacco smoking in 2004 [20] showed that men who exclusively smoked cigars and/or pipes had a higher risk of lung, tracheal and bronchial cancers than never smokers. Cancer risk was highest in people who reported inhaling cigar smoke but still 2-5 times higher among those who reported no inhalation. Subsequent systematic reviews quantified and confirmed the high excess risk of lung cancer and mortality associated with these tobacco products [12, 13]. IARC also outlined that the concentrations in smoke of nicotine, tar, carbon monoxide, and two class 1 carcinogens [benzo[*a*]pyrene and NNK: 4-(methylnitrosamino)-1-(3-pyridyl)-1-butanone] were substantially higher in cigars than cigarettes. This is mainly because cigars do not have the same filter and ventilation properties as cigarettes, making them more harmful, gram for gram.

In studies examining people who exclusively smoked cigars or pipes, there was consistent evidence of increased risks of lung cancer compared to non-smokers. Studies showed the effect on lung cancer incidence: Joly et al. [21] (relative risk RR = 4); Lubin et al. [22] (RR = 2.9 cigars only, RR = 2.5 pipes only); Iribarren et al. [23] (RR = 2.14, and RR = 3.24 for ≥5 cigars per day); and Shaper et al. [24] (RR = 5.5). While others showed the effect on lung cancer mortality: Chow et al. [25] (RR = 3.5); Henley et al. [26] (RR = 5, pipes only); Shapiro et al. [27] (RR = 5.1 cigars only); and Christensen et al. [28] (HR = 3.3 cigars only, HR = 1.5 pipes only).

Several other studies examined the effects on people who smoked both cigarettes and cigars or pipes. The effect on lung cancer incidence, compared to never-smokers, was: Lubin et al. [22] (RR = 6.9 both cigarettes and cigars, RR = 8.1 both cigarettes and pipes); Benhamou et al. [29] (RR = 9.3 for cigarette, cigar and pipe smokers); Higgins et al. [30] (RR = 10.5); Boffetta et al. [31] (RR = 12.7); McCormack et al. [32] (HR = 15.2 cigarette and cigar, HR = 14.1 cigarette and pipe); Abelin et al. [33] (RR = 5.7 for cigarette, cigar and pipe smokers).

Malhotra et al. [34] combined data on the risk of lung cancer in men who only used cigars and/or pipes from 5 large cohort studies from the US, Australia and The Netherlands, showing RR = 2.04, compared to never-smokers. Higher risks were observed among those who smoked these products and cigarettes. There were also excess risks for cancers of the head and neck (RR = 1.51), liver (RR = 1.56), oesophagus (RR = 1.29), and bladder (RR = 1.16) in those who exclusively smoked cigars or pipes.

Despite the higher concentration of toxins in cigars and pipes than cigarettes, smokers consume fewer of them and they tend to inhale less than cigarettes. This explains why the relative risks (typically 2-5) compared to never-smokers are lower than cigarette smokers (typically 20-25). There was previously a view that switching from cigarettes to cigars or pipes could substantially reduce harm. But a large prospective study [35] showed that the lung cancer mortality rate among people who switched (RR = 8.6, versus lifelong never-smoker) was noticeably higher than pipe and cigar smokers who never smoked cigarettes (RR = 3.2). Schivo et al. [36] also concluded that cigar smoking is not associated with harm reduction, compared to cigarette smoking, to make them sufficiently safe.

In the UK, there had been a rise in the number of people who smoke cigars, pipes and shisha [37]. This could impact the size of the LDCT screening-eligible population in years to come. People also use smokeless tobacco products such as snus that also contain toxins and carcinogens [38]. Given the rise in vaping and e-cigarettes, long-term studies are needed to show the impact on cancer risk, especially lung cancer.

Our report has several limitations. First, our study had smokers who were already eligible for LDCT using either USPSTF or PLCO_m2012_ criteria. Therefore, the percentage of smokers whose risk would exceed the PLCO_m2012_ or LLPv2 threshold if cigars, cigarillos or pipes were taken into account might actually be higher among the wider group of people to be initially assessed for eligibility in a screening programme, i.e. using primary care records. Second, there were only 24 lung cancers among the 511 regular users of cigars, cigarillos and pipes, such that the hazard ratios were not statistically significant. But the point estimates and findings are consistent with other studies. Third, very few SUMMIT participants who smoked cigars/pipes were women, so we are unable to show the effects of these products on lung cancer risk in this specific group. Fourth, when converting cigars and pipes into cigarette equivalents, we used a published source but acknowledge that such conversions may not be perfect given differences in the tobacco products and manufacturing process, and how they are inhaled by smokers. We did not include other products such as marijuana as it is difficult to quantify reliably (under-reporting) and it is often mixed with tobacco products. But these are also expected to contribute to lung cancer risk.

In conclusion, our contemporary cohort study confirms older evidence that cigarette smokers who also use cigars, cigarillos or pipes have a substantial risk of lung cancer. Importantly, we demonstrate that ignoring these other tobacco products when considering a person’s risk can make some ineligible for lung cancer screening. Those involved in delivering organised population screening must consider the tobacco habits of the target group and incorporate other known harmful tobacco products, not cigarettes alone, when estimating total exposure, as already intended in the UK lung cancer screening programme. This would maximise the number of high-risk individuals who can benefit from LDCT screening.

## Supplementary information

Supplementary material

Author instructions: Consortia formatting.

## Data Availability

The datasets generated and/or analysed during the current study are not yet publicly available due to the license.
