# Supplementary material for: The effect of cigar, cigarillo and pipe smoking on lung cancer risk and screening eligibility
Source: Br J Cancer. 2026 Aug 7;135(10):1641–8. doi: 10.1038/s41416-026-03578-9 (PMC13740280; doi:10.1038/s41416-026-03578-9)
Supplement: Supplementary file 1 [file 41416_2026_3578_MOESM2_ESM.pdf]

Author instructions: Consortia formatting

Introduction

The purpose of this document is to clarify the format for supplying consortia within the scope of our consortia policy. The document covers authors (individuals named separately in the main author list), consortia (a collective of individuals) and individual consortia members.

The format for supplying consortia in your paper will depend on whether one or more members of the consortia meet the criteria for authorship (for example, directly contributing to the work through data analysis, writing, and/or through data generation and curation of datasets; see our authorship guidelines http://www.nature.com/authors/policies/authorship.html for further information).

Our consortia policy states that:

- For consortia that have members that meet authorship criteria:
    - Manuscripts can have one or more consortia in the author list.
    - A consortium can be listed as an author even if not all members meet the criteria for authorship.
    - All consortium members meeting authorship criteria must be listed in the main article to ensure appropriate credit for the work and listing by third-party indexers.
    - An author may be listed in the main author list as well as in the consortium members list(s) in a separate section at the end of the paper.
    - The full list of consortium members (i.e. those who are authors and any who are not) can be placed in the Supplementary Information, as long as those who are authors are listed in the main paper (see above).
    - We allow 'on behalf of' wording in the author list. This wording can be used when some (but not all) consortium members and consortium qualify for authorship. This covers the scenario where some consortium members have contributed substantially to the data or writing process and qualify for authorship and the paper represents the results/opinion/recommendation of the consortium.
    - The affiliations of consortium members included in the paper must also be provided within the paper and their numbering must run in sequential order, following the affiliations of the main authors listed in the author list. Any affiliations repeated from the main author affiliation list should take the same number where present within a consortium.
    - Consortium members may be listed in categories or subgroups (that is, members grouped by institution, country or type of contribution/role). A member may also appear in more than one subgroup of a consortium.
    - It is also possible for consortia to have members that are other consortia. Authors and affiliations should be provided for contributing authors from the other, nested consortia also (see below for information on numbering).

- For consortia where none of the members meets authorship criteria:

- The consortium cannot be listed as an author, but can be referred to in the Acknowledgements, and the list of members placed in the Supplementary Information.

Below are instructions for the formatting of consortia. For guidelines on authorship and consortia, please visit: http://www.nature.com/authors/policies/authorship.html

Layout

Below are examples of different cases of author and consortia involvement in a paper and how the author list and consortia member list(s) should be formatted. Note that if consortia members and affiliations are very large, these may be included in the online PDF version of the paper, but not the print version. These examples are:

(1) Single consortium in the main author list (no individual authors are listed in the main author list).

(2) Single consortium and individual authors in the main author list (all consortium members have authorship status).

(3) Single consortium and individual authors in the main author list (all consortium members have authorship status) with some of the individual authors also included within the consortium.

(4) Single consortium and individual authors in the main author list (all consortium members have authorship status); equally contributing authors and present address designations included.

(5) Multiple consortia and individual authors in the main author list (all consortia members have authorship status).

(6) Multiple consortia and individual authors in the main author list (all consortia members have authorship status); nested consortia (consortia included in list of members of another consortium) and consortia subgroups included.

(7) Single consortium and individual authors in the main author list; not all consortia members have authorship status.

(8) Use of 'on behalf of' wording in the author list. The main author list comprises individual consortium members and 'on behalf of Consortium X; the consortium author list includes the individual consortium members from the main author list and additional consortium members that qualify for authorship'

(9) Use of 'on behalf of' wording in the author list. The main author list comprises individual consortium members and 'on behalf of Consortium X'; the consortium author list only includes the individual consortium members from the main author list.

If the authorship composition of your paper does not conform to any of the below examples, please contact the handling editor for advice. When re-submitting your manuscript via the online submission system, the consortium name should also be entered as an author, if it is included in the main author list, together with the contact details of a nominated consortium representative.

Example layouts for different authorship scenarios

(1) Single consortium in the main author list (no individual authors are listed in the main author list). If a consortium is the only main author listed on the first page of the paper, then the consortium authors' affiliations should be numbered sequentially starting from 1 at the end of the paper:

--- First (title) page of the paper ---

Consortium*

*A list of authors and their affiliations appears at the end of the paper.

--- Separate section at the end of the paper ---

Consortium

Consortium Author A[1,2], Consortium Author B[3] & Consortium Author C[4]

[1]Affiliation, City, Country. [2]Affiliation, City, Country. [3]Affiliation, City, Country. [4]Affiliation, City, Country.

(2) Single consortium and individual authors in the main author list (all consortium members have authorship status). If the paper contains one consortium (indicated with an asterisk) within the main author list, then the consortium author affiliations would sequentially follow the last author affiliation in the main author list (any affiliation present in both the main author list and the consortium should retain the affiliation numbering from the main author list):

--- First (title) page of paper ---

Author A[1], Author B[2], Consortium* & Author C[3]

[1]Affiliation, City, Country. [2]Affiliation, City, Country. [3]Affiliation, City, Country. *A list of authors and their affiliations appears at the end of the paper.

--- Separate section at the end of the paper ---

Consortium Name

Consortium Author a[4,5], Consortium Author b[6,7] & Consortium Author c[1,8]

[4]Affiliation, City, Country. [5]Affiliation, City, Country. [6]Affiliation, City, Country. [7]Affiliation, City, Country. [8]Affiliation, City, Country.

(3) Single consortium and individual authors in the main author list (all consortium members have authorship status) with some of the individual authors also included within the consortium. Authors from the main author list can be included within a consortium (any affiliation present in both the main author list and the consortium should retain the affiliation numbering from the main author list):

--- First (title) page of paper ---

Author A[1], Author B[2], Consortium* & Author C[3]

[1]Affiliation, City, Country. [2]Affiliation, City, Country. [3]Affiliation, City, Country. *A list of authors and their affiliations appears at the end of the paper.

--- Separate section at the end of the paper ---

Consortium Name

Consortium Author a[4,5], Consortium Author b[6,7], Consortium Author c[1,8] & Author C[3]

[4]Affiliation, City, Country. [5]Affiliation, City, Country. [6]Affiliation, City, Country. [7]Affiliation, City, Country. [8]Affiliation, City, Country.

(4) Single consortium and individual authors in the main author list (all consortium members have authorship status); equally contributing authors and present address designations included. If there are equally contributing authors and present addresses in the main author list that also contains a consortium, the present address is numbered from the end of the main author affiliations and the equally contributing authors are numbered from the end of the consortia affiliations. Furthermore, in the rare case where it is necessary to have an unaffiliated author (e.g., if the author is not associated with an institute and does not want to include a personal address), a note saying 'Unaffiliated' should be added for the author affiliation and this should be numbered at the very end of the numbering scheme:

--- First (title) page of paper ---

Author A[1,4,10], Author B[2,10], Consortium* & Author C[3,11]

[1]Affiliation, City, Country. [2]Affiliation, City, Country. [3]Affiliation, City, Country. [4]Present address: Affiliation, City, Country. [10]These authors contributed equally: Author A, Author B. [11]Unaffiliated: Author C. *A list of authors and their affiliations appears at the end of the paper.

--- Separate section at the end of the paper ---

Consortium Name

Consortium Author a[5,6], Consortium Author b[7,8] & Consortium Author c[1,9]

[5]Affiliation, City, Country. [6]Affiliation, City, Country. [7]Affiliation, City, Country. [8]Affiliation, City, Country. [9]Affiliation, City, Country.

(5) Multiple consortia and individual authors in the main author list (all consortia members have authorship status). If there are multiple consortia in the author list, the affiliation numbering for the second consortium follows on sequentially after the final affiliation number for the previous consortium:

--- First (title) page of paper ---

Author A[1], Author B[2], Consortium 1*, Consortium 2* & Author C[3]

[1]Affiliation, City, Country. [2]Affiliation, City, Country. [3]Affiliation, City, Country. *Lists of authors and their affiliations appear at the end of the paper.

--- Separate section at the end of the paper ---

Consortium 1 Name

Consortium 1 Author a[4,5], Consortium 1 Author b[6,7] & Consortium 1 Author c[1,8]

[4]Affiliation, City, Country. [5]Affiliation, City, Country. [6]Affiliation, City, Country. [7]Affiliation, City, Country. [8]Affiliation, City, Country.

Consortium 2 Name

Consortium 2 Author a[9], Consortium 2 Author b[1,10] & Consortium 2 Author c[11]

[9]Affiliation, City, Country. [10]Affiliation, City, Country. [11]Affiliation, City, Country.

(6) Multiple consortia and individual authors in the main author list (all consortia members have authorship status); nested consortia (consortia included in list of members of another consortium)

and consortia subgroups included. Authors from the main author list can be included within a consortium, multiple consortia or multiple consortia subgroups. Consortia can be nested within another consortium:

--- First (title) page of paper ---

Author A[1], Author B[2], Consortium 1*, Consortium 2* & Author C[3]

[1]Affiliation, City, Country. [2]Affiliation, City, Country. [3]Affiliation, City, Country. *Lists of authors and their affiliations appear at the end of the paper.

--- Separate section at the end of the paper ---

Consortium 1

Consortium 1 Author a[4,5], Consortium 3, Consortium 1 Author b[6] & Author A[1]

[4]Affiliation, City, Country. [5]Affiliation, City, Country. [6]Affiliation, City, Country.

Consortium 2

Subgroup 1 Consortium 2 Author a[7], Consortium 2 Author b[8] & Author C[3]

Subgroup 2 Consortium 2 Author a[7], Consortium 2 Author c[9,10] & Consortium 2 Author d[11]

[7]Affiliation, City, Country. [8]Affiliation, City, Country. [9]Affiliation, City, Country. [10]Affiliation, City, Country. [11]Affiliation, City, Country.

Consortium 3

Consortium 3 Author a[12,13,14] & Consortium 3 Author b[1,14]

[12]Affiliation, City, Country. [13]Affiliation, City, Country. [14]Affiliation, City, Country.

(7) Single consortium and individual authors in the main author list; not all consortium members have authorship status. If a consortium contains members who meet authorship criteria and those who do not, the members meeting authorship criteria should be listed in the main paper; the full list of members including those who do not meet authorship criteria can be given in the Supplementary Information if necessary. Note that this scenario could apply to the examples above:

--- First (title) page of paper ---

Author A[1], Author B[2], Consortium*, Author C[3]

[1]Affiliation, City, Country. [2]Affiliation, City, Country. [3]Affiliation, City, Country. *A list of authors and their affiliations appears at the end of the paper.

--- Separate section at the end of the paper ---

Consortium Name

Consortium Author a[4,5], Consortium Author b[6,7] & Consortium Author c[1,8]

[4]Affiliation, City, Country. [5]Affiliation, City, Country. [6]Affiliation, City, Country. [7]Affiliation, City, Country. [8]Affiliation, City, Country. A full list of members and their affiliations appears in the Supplementary Information.

(8) Use of 'on behalf of' wording in the author list where the author list comprises selected individual consortium members and 'on behalf of Consortium X'. This can be used when some (but not all) consortium members and consortium qualify for authorship. This covers the scenario where some consortium members have contributed substantially to the data or writing process and qualify for authorship and the paper represents the results/opinion/recommendation of the consortium. The full list of members including those who do not meet authorship criteria can be given in the Supplementary Information if necessary.

--- First (title) page of paper ---

Author A[1], Author B[2] & Author C[3] on behalf of Consortium*

[1]Affiliation, City, Country. [2]Affiliation, City, Country. [3]Affiliation, City, Country. *A list of authors and their affiliations appears at the end of the paper.

--- Separate section at the end of the paper ---

Consortium Name

Author A[1], Author B[2], Author C[3], Consortium Author a[4,5], Consortium Author b[6,7] & Consortium Author c[1,8]

[4]Affiliation, City, Country. [5]Affiliation, City, Country. [6]Affiliation, City, Country. [7]Affiliation, City, Country. [8]Affiliation, City, Country.

(9) Use of 'on behalf of' wording in the author list where the author list comprises all individual consortium members and 'on behalf of Consortium X'. This can be used when some (but not all) consortium members and consortium qualify for authorship. This covers the scenario where some consortium members have contributed substantially to the data or writing process and qualify for authorship and the paper represents the results/opinion/recommendation of the consortium. The full list of members including those who do not meet authorship criteria can be given in the Supplementary Information if necessary.

--- First (title) page of paper ---

Author A[1], Author B[1], Author C[3], Author D[4,5], Author E[6,7] & Author F[1,8] on behalf of Consortium*

[1]Affiliation, City, Country. [2]Affiliation, City, Country. [3]Affiliation, City, Country. [4]Affiliation, City, Country. [5]Affiliation, City, Country. [6]Affiliation, City, Country. [7]Affiliation, City, Country. [8]Affiliation, City, Country. *A list of authors and their affiliations appears at the end of the paper.

--- Separate section at the end of the paper ---

Consortium Name

Author A[1], Author B[2], Author C[3], Author D[4,5], Author E[6,7] & Author F[1,8]
