# Supplementary material for: The effect of cigar, cigarillo and pipe smoking on lung cancer risk and screening eligibility
Source: Br J Cancer. 2026 Aug 7;135(10):1641–8. doi: 10.1038/s41416-026-03578-9 (PMC13740280; doi:10.1038/s41416-026-03578-9)
Supplement: Supplementary file 2 [file 41416_2026_3578_MOESM1_ESM.docx]

**SUPPLEMENTARY APPENDIX**

**Consortium members**

Sam M Janes,^3,8^ Jennifer L Dickson,^2,3^ Carolyn Horst,^4^ Sophie Tisi,^9^ Helen Hall,^7^ Priyam Verghese,^3^ Andrew Creamer,^3^ Thomas Callender,^3^ Ruth Prendecki,^8^ Amyn Bhamani,^3,6^ Chuen Ryan Khaw,^3,8^ Mamta Ruparel,^3^ Monica Mullin,^3,5^ Tanya Patrick1, Allan Hackshaw,^1^ Laura Farrelly,^1^ Jon Teague,^1^ Anne-Marie Hacker,^1^ Esther Arthur- Darkwa,^1^ Kitty Chan,^1^ Rachael Sarpong,^1^ Malavika Suresh,^1^ Samantha L Quaife,^12^ Arjun Nair,^3,8^ Anand Devaraj^10,11^ Kylie Gyertson,^8^ Vicky Bowyer,^8^ Ethaar El-Emir,^4^ Judy Airebamen,^4^ Alice Cotton,^4^ Kaylene Phua,^4^ Elodie Murali,^4^ Simranjit Mehta,^4^ Janine Zylstra,^4^ Karen Parry-Billings,^4^ Columbus Ife,^4^ April Neville,^4^ Paul Robinson,^4^ Laura Green,^4^ Zahra Hanif,^4^ Helen Kiconco,^4^ Ricardo McEwen,^4^ Dominique Arancon,^4^ Nicholas Beech,^4^ Derya Ovayolu,^4^ Christine Hosein,^4^ Sylvia Patricia Enes,^4^ Jane Rowlands,^4^ Sheetal Karavadra,^4^ Aashna Samson,^4^ Urja Patel,^4^ Fahmida Hoque,^4^ Hina Pervez,^4^ Sofia Nnorom,^4^ Moksud Miah,^4^ Julian McKee,^4^ Mark Clark,^4^ Jeannie Eng,^4^ Fanta Bojang,^4^ Claire Levermore,^4^ Anant Patel,^13^ Sara Lock,^14^ Alan Shaw,^14^ Rajesh Banka,^15^ Angshu Bhowmik,^16^ Ugo Ekeowa,^17^ Zaheer Mangera,^18^ Chris Valerio,^18^ William M Ricketts,^19^ Neal Navani,^3,8^ Ali Mohammed,^19^ Terry O'Shaughnessy,^19^ Charlotte Cash,^13^ Magali Taylor,^8^ Samanjit Hare,^13^ Tunku Aziz,^19^ Stephen Ellis,^19^ Anthony Edey,^20^ Graham Robinson,^21^ Alberto Villanueva,^22^ Hasti Robbie,^4^ Elena Stefan,^17^ Charlie Sayer,^23^ Nick Screaton,^24^ Navinah Nundlall,^8^ Lynsey Gallagher,^8^ Andrew Crossingham,^8^ Thea Buchan,^8^ Tanita Limani,^8^ Kate Gowers,^3^ Kate Davies,^3^ John McCabe,^3^ Joseph Jacob,^3,25^ Mehran Azimbagirad,^25^ Burcu Ozaltin,^25^ Tania Anastasiadis,^26^ Andrew Perugia,^27^ James Rusius,^27^ Geoff Bellingan,^8^ Maureen Browne,^8^ Eleanor Hellier,^8^ Catherine Nestor^8^

Member affiliations (when they were involved in the study)

12. Centre for Cancer Screening, Prevention and Early Diagnosis, Wolfson Institute of Population Health, London, UK

13. Royal Free London NHS Foundation Trust, London, UK

14. Whittington Health NHS Trust, London, UK

15. Barking, Havering and Redbridge University Hospitals NHS Trust, Romford, UK

16. Homerton University Hospital Foundation Trust, London, UK

17. The Princess Alexandra Hospital NHS Trust, Harlow, UK

18. North Middlesex University Hospital NHS Trust, London, UK

19. Barts Health NHS Trust, London, UK

20. North Bristol NHS Trust, Bristol, UK

21. Royal United Hospitals Bath NHS Foundation Trust, Bath, UK

22. Surrey and Sussex Healthcare NHS Trust, Redhill/Crawley, UK

23. University Hospitals Sussex NHS Foundation Trust, Brighton, UK

24. Royal Papworth Hospital NHS Foundation Trust, Cambridge, UK

25. Satsuma Lab, Centre for Medical Image Computing (CMIC), London, UK

26. Tower Hamlets Clinical Commissioning Group, London, UK

27. Noclor Research Support, London, UK

**Supplementary Table 1. Characteristics of regular cigar, pipe, or cigarillo smokers**

|  |  | **Regularly smoked cigars or cigarillos**  **(N=381, 2.9%)** | **Regularly smoked pipes**  **(N=162, 1.2%)** | **Regularly smoked cigars / cigarillos / pipes**  **(N=511, 3.9%)** | **All SUMMIT participants**  **(N=13,000)** |
| --- | --- | --- | --- | --- | --- |
| **Lung cancer** | No | 365 (95.8%) | 154 (95.06%) | 487 (95.3%) | 12346 (94.97%) |
|  | **Yes** | **16 (4.2%)** | **8 (4.94%)** | **24 (4.7%)** | **654 (5.03%)** |
| **Sex** | Female | 34 (8.92%) | 3 (1.85%) | 36 (7.05%) | 5529 (42.53%) |
|  | Male | 347 (91.08%) | 159 (98.15%) | 475 (92.95%) | 7471 (57.47%) |
| **Ethnicity** | White | 359 (94.23%) | 147 (90.74%) | 476 (93.15%) | 11379 (87.53%) |
|  | Black | 12 (3.15%) | 3 (1.85%) | 15 (2.94%) | 681 (5.24%) |
|  | Asian/other | 10 (2.62%) | 12 (7.41%) | 20 (3.91%) | 940 (7.23%) |
| **Education** | Finished school at or before 15 years | 145 (38.06%) | 57 (35.19%) | 192 (37.57%) | 5179 (39.84%) |
|  | Completed CSCs, O-levels, or equivalent | 74 (19.42%) | 36 (22.22%) | 103 (20.16%) | 3098 (23.83%) |
|  | Completed A-levels or equivalent | 37 (9.71%) | 10 (6.17%) | 42 (8.22%) | 1398 (10.75%) |
|  | Completed further education but not a degree | 39 (10.24%) | 15 (9.26%) | 52 (10.18%) | 1093 (8.41%) |
|  | Completed a bachelor’s degree or equivalent | 63 (16.54%) | 32 (19.75%) | 90 (17.61%) | 1591 (12.24%) |
|  | Completed a further degree, e.g. masters/PhD | 23 (6.04%) | 12 (7.41%) | 32 (6.26%) | 641 (4.93%) |
| **IMD quintile** | 1 (most deprived) | 77 (20.37%) | 41 (25.47%) | 108 (21.3%) | 4145 (32.23%) |
|  | 2 | 104 (27.51%) | 37 (22.98%) | 132 (26.04%) | 3872 (30.11%) |
|  | 3 | 75 (19.84%) | 36 (22.36%) | 106 (20.91%) | 2280 (17.73%) |
|  | 4 | 89 (23.54%) | 33 (20.5%) | 117 (23.08%) | 1912 (14.87%) |
|  | 5 (least deprived) | 33 (8.73%) | 14 (8.7%) | 44 (8.68%) | 651 (5.06%) |
| **Age** | Mean (SD)  Median (IQR) | 67.5 (5.8)  68 (63-72) | 69.4 (5.3)  70 (66-73) | 68 (5.7)  69 (64-73) | 65.4 (6.1)  65 (60-70) |
| **BMI, kg/m^2^** | Mean (SD)  Median (IQR) | 28.7 (4.9)  28.2 (25.5-31.4) | 28.4 (4.8)  27.6 (25-31.3) | 28.7 (4.9)  28.1 (25.4-31.6) | 28 (5.4)  27.4 (24.3-31) |
| **Total smoking amounts a day (equal to number of cigs)** | Mean (SD)  Median (IQR) | 26.7 (11.7)  23 (20-32.4) | 24.2 (12.6)  20.2 (15.1-30) | 25.9 (11.8)  21.5 (18.2-30.8) | 21.1 (9.3)  20 (15-22.9) |
| **Cigarettes smoked a day** | Mean (SD)  Median (IQR) | 23 (11.4)  20 (17-25) | 22.2 (11.8)  20 (15-25) | 22.7 (11.4)  20 (15-25) | 21.2 (10.2)  20 (15-21) |
| **Cigar amount per week** | Mean (SD)  Median (IQR) | 31.2 (30)  25 (10-40) | 24.4 (42.5)  10 (4-25) | 31.2 (30)  25 (10-40) | 31.2 (30)  25 (10-40) |
| **Cigarillos per week** | Mean (SD)  Median (IQR) | 53.6 (38.8)  50 (20-70) | 60.8 (61.5)  37.5 (20-81) | 53.6 (38.8)  50 (20-70) | 53.6 (38.8)  50 (20-70) |
| **Pipe amount per week** | Mean (SD)  Median (IQR) | 21.2 (18.5)  14 (7-35) | 22.2 (29.8)  14 (7-35) | 22.2 (29.8)  14 (7-35) | 22.2 (29.8)  14 (7-35) |

“Regular” is defined as smoking at least once a week and includes those who are former or current smokers of these products

**Supplementary Table 2. Smoking habits of regular cigar, pipe, or cigarillo smokers, and current or former cigarette smokers**

|  |  | **Regularly smoke cigars / cigarillos & current cigarette smokers**  **(N=99, 0.8%)** | **Regularly smoke pipes & current cigarette smokers**  **(N=52, 0.4%)** | **Regularly smoke cigars / cigarillos / pipes & CURRENT cigarette smokers**  **(N=143, 1.1%)** | **Regularly smoke cigars / cigarillos / pipes & FORMER cigarette smokers**  **(N=368, 2.8%)** | **All SUMMIT participants**  **(N=13,000)** |
| --- | --- | --- | --- | --- | --- | --- |
| **Total smoking amounts a day (number of cigs or equivalent)** | Mean (SD)  Median (IQR) | 25.7 (12.5)  20.4 (17.4-30.4) | 19.3 (9.8)  17.9 (12.8-21.3) | 23.3 (11.5)  20.2 (15.4-30) | 26.8 (11.8)  23.2 (20-32.9) | 21.1 (9.3)  20 (15-22.9) |
| **Cigarettes smoked a day** | Mean (SD)  Median (IQR) | 21.9 (13.8)  20 (15-20) | 17.7 (7.7)  16 (11-20) | 20.4 (12.2)  20 (15-20) | 23.6 (10.9)  20 (18-29) | 21.2 (10.2)  20 (15-21) |
| **Cigar amount per week** | Mean (SD)  Median (IQR) | 25 (21.8)  20 (10-35) | 22 (16.9)  22.5 (10.5-33.5) | 25 (21.8)  20 (10-35) | 33.4 (32.2)  28 (10-40) | 31.2 (30)  25 (10-40) |
| **Cigarillos per week** | Mean (SD)  Median (IQR) | 54.3 (40.2)  50 (20-70) | 55 (57.4)  30 (20-90) | 54.3 (40.2)  50 (20-70) | 53.4 (38.6)  50 (20-70) | 53.6 (38.8)  50 (20-70) |
| **Pipe amount per week** | Mean (SD)  Median (IQR) | 27 (23.1)  18 (10.5-42.5) | 19 (16.2)  13.5 (7-29) | 19 (16.2)  13.5 (7-29) | 23.7 (34.3)  14 (7-35) | 22.2 (29.8)  14 (7-35) |

**Supplementary Table 3. Cigarette habits among those who smoked cigarettes only compared to current regular users of cigars, cigarillos or pipes**

|  | **Current cigar/pipe/cigarillo smokers and current cigarette smokers (n=31) *** | | **Current cigar/pipe/cigarillo smokers and former cigarette smokers (n=31) **** | | **Former cigarette smokers only (n=6272)** | | **Current cigarette smokers only (n=6217)** | |
| --- | --- | --- | --- | --- | --- | --- | --- | --- |
|  | **Mean (SD)** | **Median (IQR)** | **Mean (SD)** | **Median (IQR)** | **Mean (SD)** | **Median (IQR)** | **Mean (SD)** | **Median (IQR)** |
| Number of cigarettes per day | 23.0 (12.8) | 20 (15-25) | 23.8 (9.4) | 20 (20-30) | 22.8 (10.8) | 20 (17-25) | 19.3 (9.3) | 20 (15-20) |
| Cigarette duration, years | 50.4 (6.0) | 50 (47-54) | 37.9 (9.3) | 39 (30-44) | 42.2 (7.7) | 42 (37-47) | 47.5 (6.9) | 47 (43-52) |

These tables exclude individuals who were only occasional users of cigars, pipes, or cigarillos (i.e. less than once per week) because the impact on lung risk is likely to be minimal and information about the amount of tobacco and number of years smoking was not recorded therefore it was not possible to calculate a PLCO or LLP risk score which requires these values.
